# Cardio- and Cerebrovascular Outcomes of Postoperative Acute Kidney Injury in Noncardiac Surgical Patients With Hypertension

**DOI:** 10.3389/fphar.2021.696456

**Published:** 2021-08-27

**Authors:** Yan Guangyu, Lou Jingfeng, Liu Xing, Yuan Hong, Lu Yao

**Affiliations:** ^1^ Center of Clinical Research, The Third Xiangya Hospital, Central South University, Changsha, China; ^2^ Department of General Surgery, The Third Xiangya Hospital, Central South University, Changsha, China; ^3^ Department of Anesthesia, The Third Xiangya Hospital, Central South University, Changsha, China; ^4^ Department of Life Science and Medicine, King’s College London, London, United Kingdom; ^5^ Key Laboratory of Medical Information Research(Central South University), College of Hunan Province, Changsha, China

**Keywords:** acute kidney injury, noncardiac surgery, cardiovascular and cerebrovascular outcomes, hypertension, mortality

## Abstract

**Background:** The cardiovascular and cerebrovascular risk of postoperative acute kidney injury (AKI) in surgical patients is poorly described, especially in the hypertensive population.

**Methods**: We conducted a retrospective cohort study among all hypertensive patients who underwent elective noncardiac surgery from January 1st, 2012 to August 1st, 2017 at the Third Xiangya Hospital. The primary outcomes were fatal stroke and fatal myocardial infarction (MI). The secondary outcomes were all-cause mortality.

**Results:** The postoperative cumulative mortality within 3 months, 6 months, 1 year, 2 years, and 5 years were 1.27, 1.48, 2.15, 2.15, and 5.36%, for fatal stroke, and 2.05, 2.27, 2.70, 3.37, and 5.61% for fatal MI, respectively, in patients with postoperative AKI. Compared with non-AKI patients, those with postoperative AKI had a significantly higher risk of fatal stroke and fatal MI within 3 months [hazard ratio (HR): 5.49 (95% CI: 1.88−16.00) and 11.82 (95% CI: 4.56−30.62), respectively], 6 months [HR: 3.58 (95% CI: 1.43−8.97) and 9.23 (95% CI: 3.89−21.90), respectively], 1 year [HR: 3.64 (95% CI: 1.63−8.10) and 5.14 (95% CI: 2.50−10.57), respectively], 2 years [HR: 2.21 (95% CI: 1.03−4.72) and 3.06 (95% CI: 1.66−5.64), respectively], and 5 years [HR: 2.27 (95% CI: 1.30−3.98) and 1.98 (95% CI: 1.16−3.20), respectively]. In subgroup analysis of perioperative blood pressure (BP) lowering administration, postoperative AKI was significantly associated with 1-year and 5-year risk of fatal stroke [HR: 9.46 (95% CI: 2.85−31.40) and 3.88 (95% CI: 1.67−9.01), respectively] in patients with ACEI/ARB, and MI [HR: 6.62 (95% CI: 2.23−19.62) and 2.44 (95% CI: 1.22−4.90), respectively] in patients with CCB.

**Conclusion:** Hypertensive patients with postoperative AKI have a significantly higher risk of fatal stroke and fatal MI, as well as all-cause mortality, within 5 years after elective noncardiac surgery. In patients with perioperative administration of ACEI/ARB and CCB, postoperative AKI was significantly associated with higher risk of fatal stroke and MI, respectively.

## Introduction

Acute kidney injury (AKI) affects millions of patients worldwide, which has become an increasing health problem (Susantitaphong et al., 2013). As a major postoperative complication, AKI happens in 1.8–39.3% of all surgical patients (O'Connor et al., 2016). Despite renal function recovering in the majority of AKI cases, still, AKI has been confirmed as an important risk factor of CKD or end-stage renal disease (ESRD) (Ishani et al., 2009; Chawla and Kimmel, 2012; Kline and Rachoin, 2013), increased mortality after discharge, and longer length of stay (LOS) in all type of surgery (Kork et al., 2015; Lau et al., 2020; Gameiro et al., 2020). In addition to renal impairment, the current evidence supports the notion that acute cardiovascular damage due to AKI, as characterized as type 3 cardiorenal syndrome (Ronco et al., 2008), leads to other poor outcomes (Ronco et al., 2018). The postoperative AKI was found to be associated with an increased risk of cardiovascular events, such as myocardial infarction (MI) (Hansen et al., 2013), heart failure (James et al., 2011), and other major adverse cardiac events (MACE) (Anzai et al., 2010).

Besides, as a well-known risk factor for cardio- and cerebrovascular diseases, hypertension has also been found to increase the risk of postoperative AKI (Ikehata et al., 2016). There are 25% of noncardiac and 80% of cardiac surgical patients being found hypertensive before surgery (Dix and Howell, 2001; Haas and LeBlanc, 2004). Moreover, patients with a history of hypertension have a higher risk of cardiovascular events after AKI (Arias-Cabrales et al., 2018). Thus, it is of great importance to understand the cardiovascular prognosis in hypertensive surgical patients with postoperative AKI.

However, there is a shortage of studies on clinical outcomes, especially of cardio- and cerebrovascular events, in hypertensive patients with postoperative AKI. Therefore, in this retrospective cohort study, we sought to determine the risk of fatal stroke and fatal MI, as well as all-cause mortality, within 5 years after elective noncardiac surgery in hypertensive patients with postoperative AKI, and cardiovascular and cerebrovascular outcomes in patients with or without certain type of perioperative administration of blood pressure (BP) lowering medication.

## Methods

### Design and Setting

This was a retrospective cohort study. All patients were recruited from January 1st, 2012 to August 1st, 2017 at the Third Xiangya Hospital, Central South University, Changsha, China. It provides medical care for all residents living in central south China. The present study was following the guidelines of the Declaration of Helsinki and was approved by the Medical Ethics Committee of the Third Xiangya Hospital (Approval ID: R18030). All the participants have signed an informed consent form at admission and agreed to share their health information for medical research.

### Inclusion and Exclusion

During the period from January 1st, 2012 to August 1st, 2017, a total of 105,589 surgical patient records of elective surgery with hypertension diagnosis were retrospectively collected in the database of the Third Xiangya Hospital. Inclusions criteria were as follows: 1) age ≥18 years, 2) elective noncardiac surgery (surgery performed more than 2 days after planning the procedure), and 3) confirmed diagnosis of hypertension. Exclusion criteria were as follows: 1) severe preexisting chronic kidney disease (estimated glomerular filtration rate (EGFR) < 15 ml/min/1.73 m^2^); 2) previous renal transplant surgery or admission for renal transplant surgery, 3) no serum creatinine within 30 days before surgery; 4) admission for cardiac, obstetric surgery, and procedures of percutaneous puncture; 5) no records of resident ID card numbers or non-Hunan residents by ID card numbers; and 6) admission for multiple surgeries.

### Definition of AKI

Serum creatinine was used to identify whether the patients developed postoperative AKI or not. According to the serum creatinine criteria in the KDIGO classification, a serum creatinine increase greater than 26.5 mmol/L within 48 h or a 1.5-fold increase in serum creatinine within 7 days after surgery indicates that the patients have developed AKI (Palevsky et al., 2013). Urine output criteria were not included due to the lack of data. For each patient, records of preoperative serum creatinine measured 30 days before surgery were collected, and averaged values of those records were determined as baseline creatinine. The peak postoperative measurement of creatinine 48 h and/or 7 days after surgery was compared to baseline creatinine to assign postoperative AKI status.

### Outcomes and Definitions

The primary outcomes were fatal stroke and fatal MI within 5 years after the elective noncardiac surgery. The secondary outcomes were all-cause mortality. Time points of 3 months, 6 months, 1 year, 2 years, and 5 years were also included in the analysis of primary and secondary outcomes.

Date and cause of death were obtained through linkage to the death dataset in the Chinese National Death Surveillance (Liu et al., 2016) by matching the resident ID card numbers of each patient. In China, unique resident ID card numbers are assigned to every Chinese citizen at birth, in which the date of birth and location of birth are coded in. The dataset in the Chinese National Death Surveillance includes date and cause of death, which is electronically updated by multiple surveillance points. Causes of death were obtained as the International Classification of Diseases 10th revision (ICD-10) (Supplementary Table 1). Patients with a concurrent diagnosis of acute stroke or MI at admission were excluded from the concerned analysis.

### Covariates

Information on potential confounding factors was obtained from electronic medical records. The included covariates were gender; age; smoking status (yes or no); body mass index (BMI); BP at admission; baseline serum creatinine (an averaged value of 30 days before surgery); surgical type; concurrent diagnosis (yes or no) of diabetes, stroke, coronary heart disease (CHD), heart failure, chronic kidney disease (CKD), chronic obstructive pulmonary disease (COPD), and anemia; and perioperative administration of BP-lowering drugs (yes or no).

### Statistical Analyses

Analyses were performed using Stata® 14.0 package (Stata Corp LP, College Station, TX, United States). The cumulative incidence method was used to compute the absolute risk of all-cause mortality, fatal stroke, fatal MI, and composite cardiovascular and cerebrovascular mortality. Unadjusted and adjusted hazard ratios (HRs) of mortality were computed using a Cox proportional hazards regression model. Covariates adjusted include age, gender, BMI, BP at admission, baseline serum creatinine, comorbidities, type of surgery, and perioperative administration of BP-lowering drugs. Besides, a subgroup analysis of association between postoperative AKI and risk of fatal stroke and fatal MI within 1 year and 5 years was conducted, which was subgrouped by perioperative administration (yes or no) of angiotensin-converting enzyme inhibitor/angiotensin receptor blocker (ACEI/ARB), β-blocker, and calcium channel blocker (CCB) that is commonly used in our study (more than 10% of usage). Statistical significance was determined as *p*-value < 0.001 in group comparisons of baseline characteristics and *p*-value < 0.05 in Cox proportional hazards regression analysis.

## Results

**FIGURE 1:**
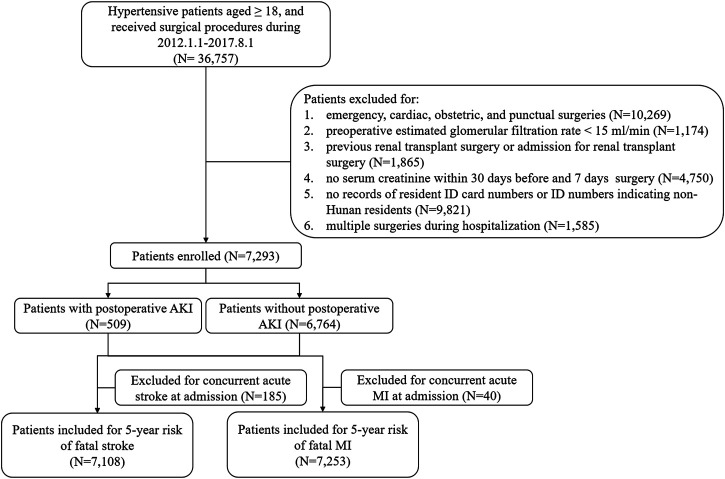
Flowchart. AKI, acute kidney injury; MI, myocardial infarction.

Postoperative episodes of AKI were found in 509 hypertensive patients (6.98%) during the first 7 postoperative days among 7,293 patients (Figure 1). Compared with non-AKI patients, AKI patients were older, more likely to be male (67.19 vs. 54.78%, *p* < 0.001), had a higher level of systolic blood pressure (SBP) (median, 137.70 vs. 132.00 mmHg, *p* < 0.001) and preoperative serum creatinine (109.00 vs. 72.00 μmol/L, *p* < 0.001), more likely to have a history of CKD (23.97 vs. 1.30%, *p* < 0.001) and anemia (34.58 vs. 8.34%, *p* < 0.001), less likely to have a history of CHD (21.81 vs. 32,95%, *p* < 0.001), less likely to have undergone neurological surgery (12.18 vs. 20.92%, *p* < 0.001), more likely to have undergone urological surgery (34.18 vs. 6.00%, *p* < 0.001), and more likely to have perioperative administration of β-blocker (42.83 vs. 30.82%, *p* < 0.001), calcium channel blocker (CCB) (56.58 vs. 41.17%, *p* < 0.001), and α-blocker (9.04 vs. 1.37%, *p* < 0.001) (Table 1).

**TABLE 1:** Baseline characteristics of hypertensive patients with or without postoperative AKI.

Variable	Postoperative AKI patients (*n* = 509)	Postoperative non-AKI patients (*n* = 6,764)	*p*-value
Age (year)	61.00 (50.00, 69.00)	56.00 (40.00, 68.00)	<0.001
Gender, male, *n* (%)	342 (67.19%)	3,705 (54.78%)	<0.001
BMI (kg/m^2^)	21.84 (19.23, 24.49)	23.42 (20.93, 25.95)	<0.001
Smoking, *n* (%)	86 (16.9%)	1,224 (18.90%)	0.500
Drinking, *n* (%)	54 (10.61%)	885 (13.09%)	0.108
SBP (mmHg)	137.70 (122.00, 151.96)	132.00 (120.00, 145.72)	<0.001
DBP (mmHg)	81.00 (71.50, 91.33)	80.00 (72.50, 88.00)	0.001
Preoperative serum creatinine (μmol/L)	109.00 (76.00, 217.41)	72.00 (58.75, 88.46)	<0.001
Comorbidities, *n* (%)			
Diabetes	100 (19.65)	1,392 (20.58)	0.616
Stroke	91 (17.88)	932 (13.78)	0.010
CHD	111 (21.81)	2,229 (32.95)	<0.001
Cardiac insufficiency	89 (17.49)	848 (12.54)	0.001
CKD	122 (23.97)	88 (1.30)	<0.001
COPD	43 (8.45)	431 (6.37)	0.067
Anemia	176 (34.58)	564 (8.34)	<0.001
Surgical type, *n* (%)			
Neurological	62 (12.18)	1,414 (20.92)	<0.001
Endocrinological	0 (0)	13 (0.19)	0.323
ENT	11 (2.16)	350 (5.17)	0.003
Respiratory	10 (1.96)	323 (4.77)	0.007
Vascular	69 (13.56)	1,136 (16.80)	0.057
Lymphatic and hemopoietic	3 (0.59)	63 (0.93)	0.436
Digestive	113(22.2)	1786 (26.39)	0.030
Urological	174 (34.18)	106 (6.00)	<0.001
Male genital	19 (3.73)	191 (2.83)	0.241
Female genital	15 (2.95)	459 (6.78)	0.001
Muscular and skeleton	29 (5.7)	530 (7.84)	0.080
Dermatological	4 (0.79)	114 (1.68)	0.123
Perioperative BP-lowering medication, *n* (%)			
ACEI/ARB	156 (30.65)	2,261 (33.43)	0.199
β-blocker	218 (42.83)	2,085 (30.82)	<0.001
CCB	288 (56.58)	2,785 (41.17)	<0.001
Diuretics	38 (7.47)	356 (5.26)	0.034
α-blocker	46 (9.04)	93 (1.37)	<0.001

AKI, acute kidney injury; BMI, body mass index; SBP, systolic blood pressure; DBP, diastolic blood pressure; CHD, coronary heart disease; CKD, chronic kidney disease; COPD, chronic pulmonary disease; ENT, ear, nose, and throat; BP, blood pressure; ACEI, angiotensin-converting enzyme inhibitor; ARB, angiotensin receptor blocker; CCB, calcium channel blocker.

All continuous variables were summarized as median (interquartile range) and tested by the Kruskal–Wallis test.

All categorical variables were summarized as number (proportion) and tested by chi-square test.

**FIGURE 2:**
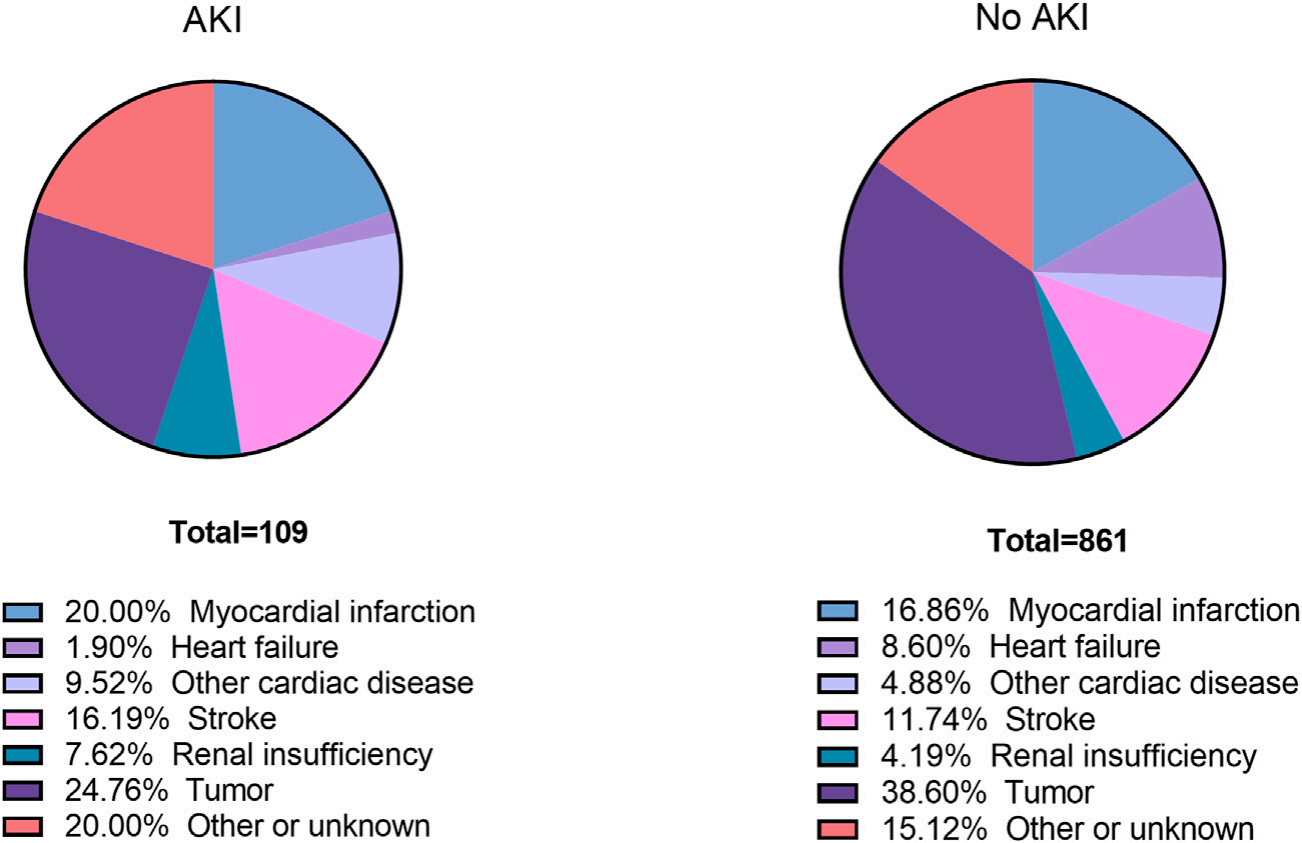
Causes of death by postoperative AKI status. AKI, acute kidney injury.

Total follow-up time was 400,802 person-months with a duration of 5 years (Supplementary Table 2). 970 patients died during the 5 years (109 AKI patients and 861 non-AKI patients) (Supplementary Table 3). The specific cause of death within 5 years after elective noncardiac surgery is shown in Figure 2. MI was found as the cause of death in 20.00% of AKI patients and 16.86% of non-AKI patients. Stroke was found as the cause of death in 16.19% of AKI patients and 11.74% of non-AKI patients.

### Fatal Stroke and Fatal MI After Elective Noncardiac Surgery

165 non-AKI patients and 20 AKI patients were excluded due to the diagnosis of concurrent acute stroke at admission when analyzing the risk of fatal stroke after elective noncardiac surgery. And 33 non-AKI patients and 7 AKI patients were excluded due to the diagnosis of concurrent acute MI at admission in the analysis for risk of fatal MI.

**FIGURE 3:**
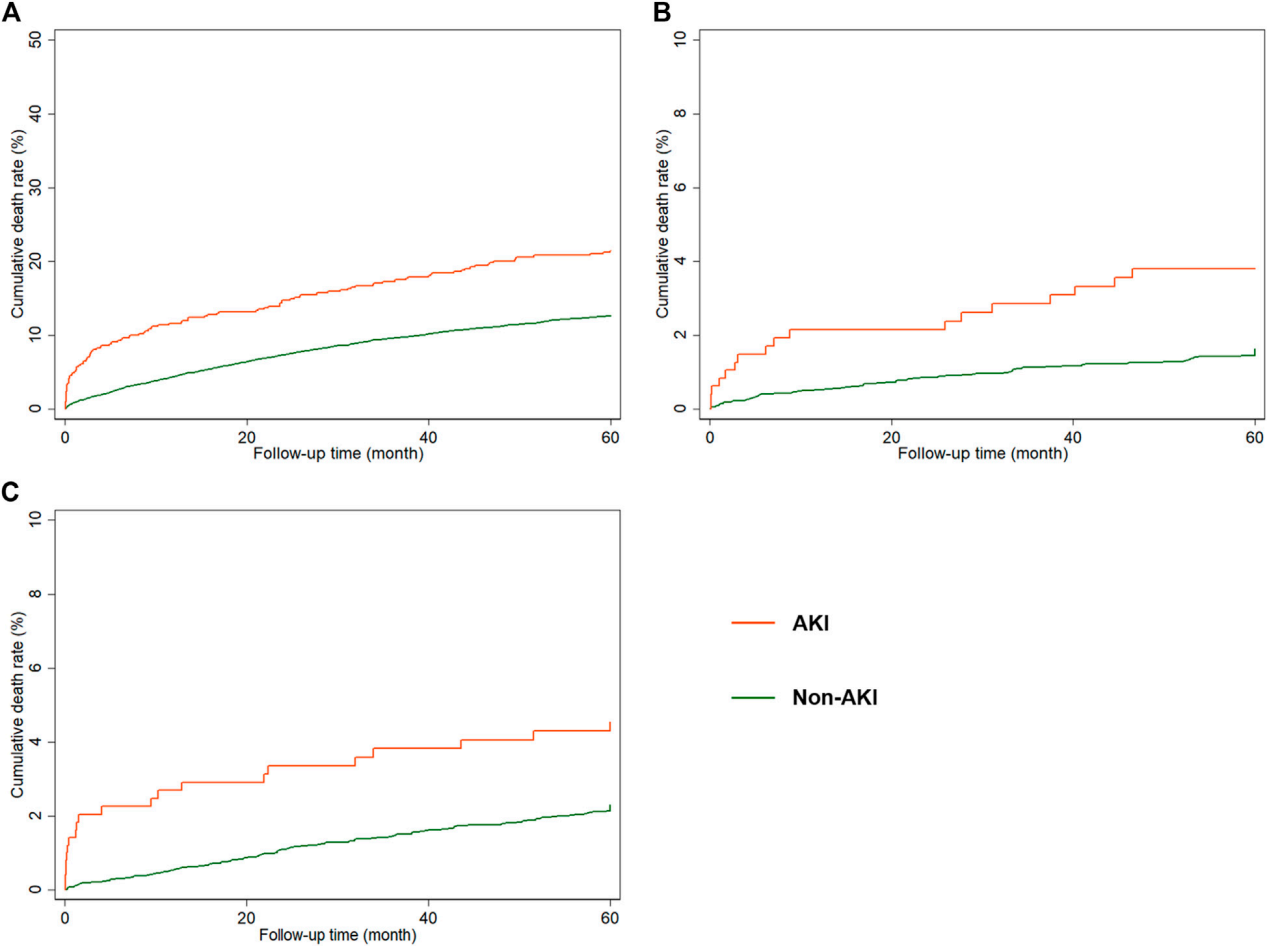
Kaplan–Meier curves for cumulative risks of 5-year mortality. (**A)** Cumulative risk of all-cause mortality. (**B)** Cumulative risk of fatal MI. (**C)** Cumulative risk of fatal stroke. AKI, acute kidney injury.

Kaplan–Meier curves showed that patients with postoperative AKI have higher cumulative risks of fatal stroke and fatal MI within 5 years (Figure 3). Within 3 months, 6 months, 1 year, 2 years, and 5 years after surgery, the cumulative risks of fatal stroke were 1.27, 1.48, 2.15, 2.15, and 5.36%, respectively, among AKI patients, and 0.23, 0.41, 0.52, 0.86, and 2.33%, respectively, among non-AKI patients (Supplementary Table 3). And the cumulative risks of fatal MI were 2.05, 2.27, 2.70, 3.37, and 5.61% among AKI patients, and 0.19, 0.28, 0.54, 1.08, and 3.42% among non-AKI patients, at the corresponding time points mentioned above (Supplementary Table 3).

**FIGURE 4:**
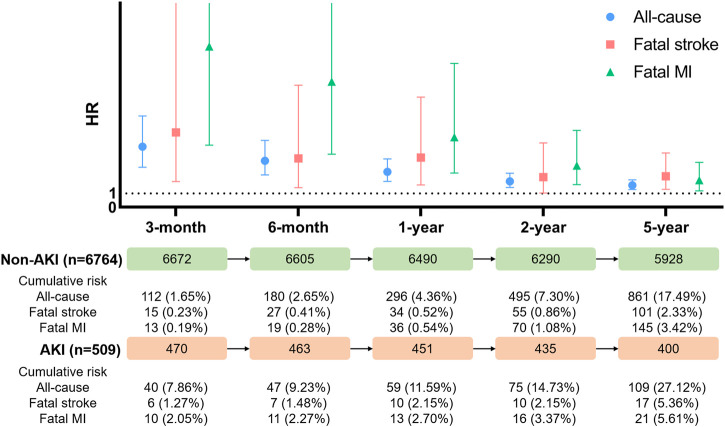
Hazard ratio for all-cause mortality, fatal stoke, and fatal myocardial infarction. AKI, acute kidney injury; MI, myocardial infarction; HR, hazard ratio.

Kaplan–Meier curves for cumulative risks of fatal stroke and fatal MI are shown in Figure 3. HRs for all outcomes were decreased by the follow-up time from 3 months to 5 years (Figure 4). The fully adjusted HRs of fatal stoke for AKI patients compared with non-AKI patients were 5.49 (95% CI: 1.88−16.00, *p* = 0.002) within 3 months, 3.58 (95% CI: 1.43−8.97, *p* = 0.006) within 6 months, 3.64 (95% CI: 1.63−8.10, *p* = 0.002) within 1 year, 2.21 (95% CI: 1.03−4.72, *p* = 0.041) within 2 years, and 2.27 (95% CI: 1.30−3.98, *p* = 0.004) within 5 years (Figure 4 and Table 2). The fully adjusted HRs of fatal MI for AKI patients compared with non-AKI patients were 11.82 (95% CI: 4.56−30.62, *p* < 0.001) within 3 months, 9.23 (95% CI: 3.89−21.90, *p* < 0.001) within 6 months, 5.14 (95% CI: 2.50−10.57, *p* < 0.001) within 1 year, 3.06 (95% CI: 1.66−5.64, *p* < 0.001) within 2 years, and 1.93 (95% CI: 1.16−3.20, *p* = 0.011) within 5 years (Figure 4 and Table 2). There were closer associations between postoperative AKI and fatal MI within 2 years than fatal stroke (Figure 4).

**TABLE 2:** Cox proportional hazard regression analysis of association between postoperative AKI and all-cause mortality, fatal stroke, and fatal MI risk in hypertensive patients after elective surgery.

Follow-up time	Unadjusted			Adjusted^a^		
	HR	95% CI	*p*-value	HR	95% CI	*p*-value
3 Months						
All-cause mortality (*n* = 7,293)	4.89	3.41-7.03	<0.001	4.44	2.93-6.71	<0.001
Fatal stroke (*n* = 7,108)	5.60	2.17-14.42	<0.001	5.49	1.88-16.00	0.002
Fatal MI (*n* = 7,253)	10.72	4.70-24.44	<0.001	11.82	4.56-30.62	<0.001
6 Months						
All-cause mortality (*n* = 7,293)	3.65	2.65-5.03	<0.001	3.41	2.37-0.4.90	<0.001
Fatal stroke (*n* = 7,108)	3.67	1.60-8.43	0.002	3.58	1.43-8.97	0.006
Fatal MI (*n* = 7,253)	8.13	3.87-17.09	<0.001	9.23	3.89-21.90	<0.001
1 Year						
All-cause mortality (*n* = 7,293)	2.74	2.07-3.62	<0.001	2.59	1.89-3.55	<0.001
Fatal stroke (*n* = 7,108)	4.19	2.07-8.48	<0.001	3.64	1.63-8.10	0.002
Fatal MI (*n* = 7,253)	5.01	2.66-9.42	<0.001	5.14	2.50-10.57	<0.001
2 Years						
All-cause mortality (*n* = 7,293)	2.16	1.69-2.75	<0.001	1.89	1.44-2.49	<0.001
Fatal stroke (*n* = 7,108)	2.65	1.35-5.21	0.005	2.21	1.03-4.72	0.041
Fatal MI (*n* = 7,253)	3.29	1.91-5.66	<0.001	3.06	1.66-5.64	<0.001
5 Years						
All-cause mortality (*n* = 7,293)	1.82	1.49-2.22	<0.001	1.56	1.25-1.95	<0.001
Fatal stroke (*n* = 7,108)	2.45	1.46-4.09	0.001	2.27	1.30-3.98	0.004
Fatal MI (*n* = 7,253)	2.11	1.34-3.34	0.001	1.93	1.16-3.20	0.011

a Adjusted for age, gender, body mass index, preoperative creatinine, comorbidities, blood pressure at admission, location of surgery, and perioperative administration of blood pressure lowering drugs.

HR, hazard ratio; CI, confidence interval; MI, myocardial infarction.

### All-Cause Mortality and Composite Cardiovascular and Cerebrovascular Mortality After Elective Noncardiac Surgery

There were 7,293 patients included in the analysis of all-cause mortality. Kaplan–Meier curves showed that patients with postoperative AKI have higher cumulative risks of all-cause mortality within 5 years (Figure 3). The cumulative risks of all-cause mortality within 3 months, 6 months, 1 year, 2 years, and 5 years among AKI patients were 7.86, 9.23, 11.59, 14.73, and 27.12%, respectively, compared with 1.65, 2.65, 4.36, 7.30, and 17.49%, among non-AKI patients (Supplementary Table 3).

The fully adjusted HRs were 4.44 (95% CI: 2.93−6.71, *p* < 0.001), 3.41 (95% CI: 2.73−4.90, *p* < 0.001), 2.59 (95% CI: 1.89−3.55, *p* < 0.001), 1.89 (95% CI: 1.44−2.49, *p* < 0.001), and 1.56 (95% CI: 1.25−1.95, *p* < 0.001) within 3 months, 6 months, 1 year, 2 years, and 5 years, respectively (Table 2).

### Subgroup Analysis of Association Between Postoperative AKI and Risk of Fatal Stroke and Fatal MI by Perioperative Administration of ACEI/ARB, β-blocker, and CCB

Significance of association between postoperative AKI and 1-year risk of fatal stroke was found in patients only with perioperative administration of ACEI/ARB (HR: 9.46, 95% CI: 2.85−31.40) (Table 3). However, significance of associations between postoperative AKI and 1-year risk of fatal MI was found in patients with perioperative administration of ACEI/ARB (HR: 4.03, 95% CI: 1.36−11.97), β-blocker (HR: 7.23, 95% CI: 2.43−21.55), and CCB (HR: 6.62, 95% CI: 2.23−19.62) (Table 3).

**TABLE 3:** Subgroup analysis of association between postoperative AKI and risk of fatal stroke and fatal MI in 1-year and 5-years by perioperative administration of ACEI/ARB, β-blocker, and CCB.

Outcomes	Follow-up time	With administration			Without administration		
		HR^a^	95% CI	*p*-value	HR^a^	95% CI	*p*-value
Fatal stroke	1 year						
	ACEI/ARB	9.46	2.85-31.40	<0.001	3.19	1.05-9.66	0.040
	β-blocker	4.28	0.93-19.60	0.061	4.10	1.58-10.63	0.004
	CCB	1.78	0.47-6.74	0.393	6.40	2.23-18.36	0.001
	5 years						
	ACEI/ARB	3.88	1.67-9.01	0.002	1.15	0.68-3.35	0.308
	β-blocker	1.38	0.42-4.46	0.594	2.64	1.38-5.05	0.003
	CCB	2.19	0.97-4.94	0.058	2.02	0.91-4.47	0.082
Fatal MI	1 year						
	ACEI/ARB	4.03	1.36-11.97	0.012	8.36	3.00-23.25	<0.001
	β-blocker	7.23	2.43-21.55	<0.001	3.87	1.22-12.29	0.022
	CCB	6.62	2.23-19.62	0.001	5.34	1.97-14.48	0.001
	5 years						
	ACEI/ARB	1.78	0.83-3.82	0.140	1.95	0.99-3.86	0.055
	β-blocker	1.64	0.73-3.68	0.231	1.89	0.95-3.76	0.069
	CCB	2.44	1.22-4.90	0.012	1.64	0.76-3.58	0.210

a Adjusted for age, gender, body mass index, preoperative creatinine, comorbidities, blood pressure at admission, location of surgery, and perioperative administration of blood pressure lowering drugs.

AKI, acute kidney injury; ACEI, angiotensin converting enzyme inhibitor; ARB, angiotensin receptor blocker; CCB, calcium channel blocker. HR, hazard ratio; CI, confidence interval; MI, myocardial infarction.

Moreover, in patients without perioperative administration of ACEI/ARB (HR: 3.19, 95% CI: 1.05−9.66), β-blocker (HR: 4.10, 95% CI: 1.58−10.63), and CCB (HR: 6.40, 95% CI: 2.23−18.36), postoperative AKI also significantly increased the 1-year risk of fatal stroke (Table 3). Likely, significance of association between postoperative AKI and 1-year risk of fatal MI was found in patients without perioperative administration of ACEI/ARB (HR: 3.19, 95% CI: 1.05−9.66), β-blocker (HR: 4.10, 95% CI: 1.58−10.63), and CCB (HR: 6.40, 95% CI: 2.23−18.36) (Table 3).

Meanwhile, postoperative AKI significantly increased the 5-year risk of fatal stroke in patients with ACEI/ARB (HR: 3.88, 95% CI: 1.67−9.01), and without perioperative administration of β-blocker (HR: 2.64, 95% CI: 1.38−5.05) (Table 3). While it only increased the 5-year risk of fatal MI in patients with perioperative administration of CCB (HR: 2.44, 95% CI: 1.22−4.90) (Table 3).

## Discussion

As far as we know, this is the first study in hypertensive patients, which concentrates on the association between postoperative AKI and 5-year all-cause mortality, fatal stroke, and fatal MI after elective noncardiac surgery. In the current retrospective cohort study, 6.98% of adult elective noncardiac surgical patients with hypertension and without preexisting severe kidney impairment developed AKI. Compared with fatal MI, fatal stroke had a higher cumulative risk within 2 years after elective noncardiac surgery. Besides, there was a closer association between postoperative AKI and fatal MI within 2 years than that between postoperative AKI and fatal stroke. In patients with perioperative administration of ACEI/ARB, postoperative AKI was significantly associated with 1-year and 5-year risk of fatal stroke. And in patients with perioperative administration of CCB, postoperative AKI was significantly associated with 1-year and 5-year risk of fatal MI. Our findings should encourage more concerns on prevention strategies for hypertensive patients who develop reductions in kidney function after surgery.

Prognosis after postoperative AKI in hypertensive patients is not well described in previous studies that only included the general population. Patients with AKI have a 2-fold risk of death and a 2-day longer LOS when undergoing all types of surgery, and a 5-fold risk of death and a 3-day longer LOS when undergoing noncardiac surgery, than normal patients (Kork et al., 2015). In a pooled analysis including 1,817,999 participants from 70 studies, long-term risk of death was found to be associated with AKI, with the pooled crude mortality of an additional risk of 5.93 deaths per 100 years (See et al., 2019). In a British study concentrating on mortality 1 year after major noncardiac surgery, AKI was associated with an adjusted HR for death of 2.96 (95% CI: 1.86−4.71) (O'Connor et al., 2017). Also, the mortality climbs with the severity of AKI, in which a 37.1 times higher risk of death was found in patients who need dialysis after experiencing postoperative AKI (Lau et al., 2020).

This study also indicated that postoperative AKI was found to be associated with an increased risk of 5-year fatal stroke and fatal MI in hypertensive patients. Multiple studies have been conducted exploring the association between AKI and long-term cardiovascular events in the general population. In a prospective study of cardiac surgery (Hansen et al., 2013), postoperative AKI was found to be associated with a higher risk of 5-year all-cause mortality (26.5 vs. 12.1%) and insignificant increased risk of MI (5.0 vs. 3.3%). In contrary to our study, they did not find an association with the risk of stroke, which can be explained by less hypertensive subjects (56.8% in the total cohort). However, in 2017, a meta-analysis of 25 studies involving 254,408 adults confirmed that AKI associates with an elevated risk of cardiovascular mortality (RR: 1.86, 95% CI: 1.72−2.01), major cardiovascular events (RR: 1.38, 95% CI: 1.23−1.55), acute MI (RR: 1.40, 95% CI: 1.23−1.59), and stroke (RR: 1.15, 95% CI: 1.03−1.28) (Odutayo et al., 2017), which supports the result in our study.

Our finding indicated that postoperative AKI had a higher incidence of fatal MI than stroke. The current understanding of pathophysiology may explain the elevated risk of cardiovascular events after AKI (Legrand and Rossignol, 2020). AKI increases certain circulating inflammatory mediators in animal models, which exert direct cardiodepressant effects (Dépret et al., 2017; Bartekova et al., 2018). In human studies, neutrophil gelatinase–associated lipocalin (NGAL), as a predictor of AKI, was reported to be associated with the development of cardiac remodeling and vascular pro-fibrosis (Tarjus et al., 2015; Martínez-Martínez et al., 2017). Still, the pathophysiology of cardiovascular damage after acute kidney injury needs further exploration.

In subgroup analysis in our study, postoperative AKI was significantly associated with 1-year and 5-year risk of fatal stroke, as well as 1-year risk of fatal MI, in patients with perioperative administration of ACEI/ARB. In patients with perioperative administration of CCB, postoperative AKI was significantly associated with 1-year and 5-year risk of fatal MI. And postoperative AKI was significantly associated with only 1-year risk of fatal MI in patients with perioperative β-blocker. Studies reported that perioperative use of ACEI/ARB was associated with an increased incidence of intraoperative hypotension (Hollmann et al., 2018) and resulted in increased odds of postoperative AKI and mortality (Yacoub et al., 2013). A meta-analysis indicated that perioperative β-blocker was associated with an increased incidence of bradycardia (OR: 3.49; 95% CI: 2.4−5.9) and congestive heart failure (OR: 1.68; 95% CI: 1.00−2.8) after surgical procedure (Beattie et al., 2008). CCB is considered as a safe agent that can be continued during the perioperative period (Sanders et al., 2019). However, few studies focused on the association between CCB and postoperative outcomes, which were inadequate in patients with postoperative AKI.

Limitations of our work to date include the following. First, 1-year mortality is relatively low (4.98%) in our study, compared with the previous British study (7.5%) (O'Connor et al., 2017), which can be explained by the misreported deaths in the Chinese National Death Surveillance. Second, due to the limitation of the enrollment, the prognosis of postoperative AKI between hypertensive and normotensive patients did not be compared in this study. Third, patients with missing data in our study might increase bias in the subject’s enrollment.

## Conclusion

Hypertensive patients with postoperative AKI have a significantly higher risk of fatal stroke and fatal MI, as well as all-cause mortality, within 5 years after elective noncardiac surgery. In patients with perioperative administration of ACEI/ARB and CCB, postoperative AKI was significantly associated with higher risk of fatal stroke and MI, respectively.

## Data Availability

The raw data supporting the conclusion of this article will be made available by the authors, without undue reservation.
