# Supplementary material for: Cardio- and Cerebrovascular Outcomes of Postoperative Acute Kidney Injury in Noncardiac Surgical Patients With Hypertension
Source: Front Pharmacol. 2021 Aug 27;12:696456. doi: 10.3389/fphar.2021.696456 (PMC8430207; doi:10.3389/fphar.2021.696456)
Supplement: Supplementary file 1 [file Table1.DOCX]

Table S1. Identification of causes of death.

| Outcome | ICD-10 code |
| --- | --- |
| Myocardial infarction | I21 |
| Heart failure | I50 |
| Other cardiac diseases | I00-20, I23-24, I26-49, I51-52 |
| Stroke | I61, I63, I64 |
| Renal insufficiency | N17-19 |
| Tumor | C00-95, D46-47 |
| Other or unknown | All other codes not included above or unknown |

Table S2. Total follow-up time of 5 years after surgery by outcomes.

| Outcome | Patients included in analysis (n) | Total follow-up time (person-months) |
| --- | --- | --- |
| All-cause mortality | 7,293 | 400,802 |
| Fatal stroke | 7,108 | 349,073 |
| Fatal MI | 7,253 | 355,228 |

MI, myocardial infarction.

Table S3. Cumulative risk of all-cause mortality, fatal stroke, and fatal MI risk in hypertensive patients after elective surgery.

| Outcomes | Cumulative risk by follow-up time | | | | |
| --- | --- | --- | --- | --- | --- |
|  | 3 months | 6 months | 1 year | 2 years | 5 years |
| AKI, n (%) |  |  |  |  |  |
| All-cause mortality (n=509) | 40 (7.86%) | 47 (9.23%) | 59 (11.59%) | 75 (14.73%) | 109 (27.12%) |
| Fatal stroke (n=489) | 6 (1.27%) | 7 (1.48%) | 10 (2.15%) | 10 (2.15%) | 17 (5.36%) |
| Fatal MI (n=502) | 10 (2.05%) | 11 (2.27%) | 13 (2.70%) | 16 (3.37%) | 21 (5.61%) |
| Non-AKI, n (%) |  |  |  |  |  |
| All-cause mortality (n=6,784) | 112 (1.65%) | 180 (2.65%) | 296 (4.36%) | 495 (7.30%) | 861 (17.49%) |
| Fatal stroke (n=6,619) | 15 (0.23%) | 27 (0.41%) | 34 (0.52%) | 55 (0.86%) | 101 (2.33%) |
| Fatal MI (n=6,751) | 13 (0.19%) | 19 (0.28%) | 36 (0.54%) | 70 (1.08%) | 145 (3.42%) |
| All, n (%) |  |  |  |  |  |
| All-cause mortality (n=7,293) | 152 (2.08%) | 227 (3.11%) | 355 (4.98%) | 570 (7.82%) | 970 (18.17%) |
| Fatal stroke (n=7,108) | 40 (1.27%) | 47 (1.48%) | 59 (2.15%) | 75 (2.15%) | 109 (5.36%) |
| Fatal MI (n=7,253) | 40 (2.05%) | 47 (2.27%) | 59 (2.70%) | 75 (3.37%) | 109 (5.61%) |

AKI, acute kidney injury. MI, myocardial infarction.
